# Persistence of hepatitis B surface antibody to hepatitis B vaccine among medical college students in Najran University, Saudi Arabia

**DOI:** 10.6026/97320630018617

**Published:** 2022-07-31

**Authors:** Alshehri Ahmad A., Alshamrani Saleh A., Elnoubi Osman A., Alshahrani Abdullah J., Saif Ahmad M., Nahari Mohammed H., Elfaki Nahid K., Alshahrani Mohammed A., Alfateh Saeed M., Mashraqi Mutaib M.

**Affiliations:** 1 Department of Clinical Laboratory Sciences, College of Applied Medical Sciences, Najran University, Saudi Arabia; 2 Department of Public Health, General Directorate of Health Affairs in Asir Region, Abha, Saudi Arabia; 3 Department of Community Health Nursing, College of Nursing, Najran University, P.O. Box 1988, Najran, Saudi Arabia

**Keywords:** Hepatitis B virus, ABO/Rh blood groups, Chemiluminescence assay

## Abstract

Around 257 to 291 million people worldwide are infected with the hepatitis B virus (HBV). Immunization is one of the most effective ways to combat HBV infection. Saudi Arabia implemented a mandatory hepatitis B immunization program in 1989. This project investigated the levels of hepatitis B surface antibodies (anti-HBs) among medical students in the college of applied medical science at Najran University in December 2020. Students (n=82) were tested for anti-HBs levels using a chemiluminescent microparticle immunoassay (CMIA). Anti-HBs levels were the main outcome measures. Results showed that about 81.7% of participants had an insufficient amount of Anti-HBs levels (<10 IU/ L) compared to 18.3 % of participants who had protective levels of anti-HBs (≥10 IU/ L). However, 78.5% of the reactive group was at risk of losing immunity with a level between 12 and 42 IU/ L. Our study also showed an association between the age and the level of anti-HBs. Moreover, male students were at more risk than female students. Our results showed a strong relationship between Blood Groups and Anti-HBs antibody levels. The O+ (with 38.8%) and A+ (with 25.4%) blood groups showed the highest percentage amongst participants who had inadequate protection of anti-HBs (<10 IU/ L). Thus, data helps in our understanding and observations on anti-HBV immunity in individuals twenty years after being vaccinated as a child. According to the findings of our study, a large majority of students had a non-protective anti-HBs titer.

## Background:

Hepatitis B virus (HBV) infection remains an important global health issue [1-2] impacting an estimated 257-291 million individuals. Because of its clinical consequences, such as liver cirrhosis and hepatocellular cancer, HBV is linked to significant morbidity and death [3-4]. HBV, a member of the Hepadnaviridae family, is composed of enveloped protein and small, double-stranded DNA [5, 6 & 7]. The replicative cycle of the HBV genome involves an RNA intermediate that can integrate into the host genome and then survive in the infected cells, leading to HBV persistence [7]. HBV infection can be accurately diagnosed using a combination of clinical, biochemical, histological, and serologic results [8-9]. After HBV is contracted, the patient's blood may be examined for a number of viral antigens and their matching antibodies. An active (chronic or acute) viral infection, for instance, is indicated by the presence of the hepatitis B surface antigen (HBsAg) [8-9]. When the hepatitis B e antigen (HBeAg) is present, it denotes a high level of virus replication and contagiousness. These indicators are also used to assess how well patients respond to HBV treatment options. Anti-HBs is also a sign of immunity and its sero positivity is used as evidence for immunological response due to either past HBV infection or vaccination. Anti-HBs titer levels are used to evaluate the effectiveness of vaccination and the need for revaccination [8-9].

Two strategies have been shown to significantly reduce the frequency of HBV infections. The first is the introduction of HBV vaccination programs for newborns, children, adolescents, and high-risk groups. The second the is promotion of improvements in blood product screening and safe transfusion practices [10]. Following the 1989 implementation of an HBV pediatric immunization program [10-11], Saudi Arabia has shown a considerable decline in the prevalence of HBV over the past 20 years. In 1990, a vaccination booster program was introduced for children starting school, for healthcare professionals, and for other high-risk populations [10- 11]. In several nations, the effectiveness of immunization campaigns in triggering anti-HBs has been assessed. Anti-HBs levels were found to be not high enough in sizable sections of the assessed populations to reduce the risk of HBV infection [11-12].

The ABO blood group system, the most extensively investigated erythrocyte antigen system, is widely used in clinical practice and influences host susceptibility [13]. As an easily accessible factor in an individual's genetic makeup, ABO blood groups have been statistically and biologically associated with many chronic diseases as well as some viral infections [13-14]. The epidemiological studies have explored the relationship between ABO blood group and HBV infection [15]. However, the results have found that HBV prevalence in a highly endemic area was lower in blood group B, but higher in blood group O. This suggested that "universal" blood group O was associated with increased susceptibility to HBV infection [15] and precautionary measures should be taken for safe blood transfusion particularly in a community with low HBV vaccine uptake. Therefore, it is of interest to evaluate the anti-HBs levels in the serum of medical sciences students born after 2000 in order to verify the persistence of immunity over time after vaccination. It is also of interest to illustrate the correlation between the level of anti HBs-Ab and ABO/Rh blood groups.

### Subjects:

Random sampling study was carried out on students (N= 82) recruited from medical sciences colleges at Najran university. Written informed consent was obtained from all subjects after receiving an explanation of the study. For each subject, we used structured questionnaire and recorded the following demographic data: age, gender, ABO and Rhesus blood group, and date of completing HBV vaccine series during childhood. Any subject ohwreceived further boosters were excluded from the study.

### Sample handling:

5 ml venous blood were collected in plain tubes, left to clot at room temperature for 20-30 minutes, centrifuged at 4,000 x g for 5 minutes. The resulting serum was collected, aliquoted into 1.5 ml tubes and frozen at -20°C until used for antibody testing. Serum with hyperlipemia, hemolysis, and contamination was discarded.

### Chemiluminescence assay:

The architect HBsAg assay was used in the current study for the quantitative determination of hepatitis anti-HBs in human serum samples. The assay is based on sandwich Electro Chemi Luminescence immunoassay (ECLI) technology. The assay kit was obtained from Roche Diagnostics (Mannheim, Germany). The overall sensitivity was estimated to be 100 % among vaccines samples. The overall specificity of the assay was estimated to be 99.78% among blood donors. The assay was performed according to the manufacturer manual using the ARCHITECT Cobas e411 system (Roche Diagnostics, Germany). If the concentration of the specimen was greater than or equal to 10 IU/L, the specimen was considered reactive for anti-HBs; if it is less than 10 IU/L it was considered non-reactive. In addition, qualitative determination of IgG and IgM antibodies to the hepatitis B core antigen in serum sample was conducted to discriminate between immune response against infection (past or current) and against vaccination. The assay is based on the competition ECLI principle and performed according to the manufacturer manual using previously mentioned system. All tests were conducted at Najran University's hospital.

### Statistical analysis:

All the calculations and statistical analysis were performed using the lacitsitatS Package for Social Sciences, version 22 (IBM Corp, Armonk, NY, USA) and Graph Pad Prism statistical software (version 5, USA). Data were expressed as mean and standard deviation (SD). Comparisons of the anti-HBs Ab concentrations with sex, age and ABO blood grouping was determined by the Chisquare test. Results were considered statistically significant at a pvalue <0.05. Analysis that involved a single independent variable was performed using one-way ANOVA.

## Results:

A total of 82 Saudi Applied Medical Science students from Najran area, 50 male and 32 female, were enrolled in this study. All participants received the primary HBV vaccine during infancy only as shown in Table 1(see PDF). Anti-HBs levels were measured for all participants to verify the effectiveness of that vaccine to induce the humoral immune response, which showed that about 81.7% of participants, despite completing a primary HBV vaccination course during their childhood, had inadequate protection of anti-HBs (<10 IU/ L). In addition our findings revealed that about 18.3% of participants had levels of Anti-HBs at ≥ 10 IU/L in addition to that female students were more persistent in immunity compared to male students as shown in Table 2(see PDF). Interestingly about 78.5% of the reactive group had an immunity level between 12 and 42 IU/ L of anti-HBs (Figure 1 - see PDF) indicating that they are susceptible to losing immunity with time. The vast majority, unexpectedly, of this cohort were carrying the O+ blood group. In order to see the effect of aging on the level of Anti-HBs, the participants were divided into two groups. Out of 81 participants, 87.65% were aged from 20 to 24 years(group A),while12.35% were aged between 25 and 28 years (group B).Most of the participants with in group A and group B lost their immunity against HBV (85.9% and 60% respectively). Indicative but not conclusive, group A showed more persistence of immunity against HBV than group B as shown in Table 3(see PDF). Our investigation indicated an association between gender and the persistence level of Anti-HBs. Females interestingly showed persistence in immunity more than males with 25% and 14.0% respectively (Table 4 - see PDF). The relation between different ABO/Rh blood groups for the participants and persistence of immunity against HBV has been checked. Results showed that individuals with blood groups O+ (with 38.8%), A+ (with 25.4%), and O- (with 20%) had inadequate levels of immunity against HBV compared to other blood groups (Table 5 and Table 6 - see PDF). Interestingly, no participant within the O- group showed persistence of immunity over 20 year period.

## Discussion:

Infection prevention is still a top focus for individuals working or being prepared to work in the health sector. The Health authorities' goal of keeping those individuals resistant to infectious diseases is being practiced through a precautionary and management plan. Immunization has always been one of the best protective measures, as a critical way to fulfill this goal. This study examined the levels of anti-HBs of students at applied medical college at Najran University, Saudi Arabia. Our findings revealed that about 81.7% of participants, despite completing primary HBV vaccination course during their childhood, had inadequate protection of anti-HBs (<10 IU/ L). Recently local reported study showed that 75.7% of community participants aged from 18-28 Years had lost protective immunity against HBV after completion of the primary HBV vaccination series during infancy [16]. Another local study showed similar finding in which 51% of medical students in Taibah university lost protective immunity against HBV after 20 years of vaccination [17]. According to another research from Egypt, roughly 40% of school-children who completed the first HBV vaccination series had insufficient protection [18]. Furthermore, past research from Europe [18], Taiwan [19], and the United States [20] have all reported similar findings in which that anti-HBs level decreased over time. Nevertheless, about 18.3 % of participants showed immunity to HBV with anti-HBs (≥10 IU/ L). Interestingly about 78.5% of the reactive group was on the borderline of the immunity level with 12-42 IU/ L of anti-HBs indicating that they are susceptible to losing immunity with time. Our study also showed the association between age group and the level of anti-HBs. Students were categorized into two different age groups; A, (20-24 years) and B, (25-28 years). Group A showed a 65.59% with a low level of anti-HBs (≤10 IU/ L), and this result was consistent with several results that showed reduced levels of antibodies as age increases [21, 22 & 23]. Previous research has found gender differences in the response to the HBV vaccination [21]. Female students had a stronger response than male students, similar to other research that found males had lower anti-HBs antibody titers than females. This suggests that the female gender is a predictor of anti-HBs status. Therefore, the opposing actions of the sex hormones (androgen and estrogen) could cause gender differences [25]. In this study for the first time, to the best of our knowledge, our results showed a strong relationship between ABO/Rh blood groups and Anti-HBs antibody levels. The O+ (with 38.8%) and A+ (with 25.4%) blood groups showed the highest percentage amongst participants who had inadequate protection of anti-HBs (<10 IU/L). Yet, no published study has looked into this. However, a study reviewed the literature on the distribution and frequency of the ABO and Rh blood types in Saudi Arabia, highlighting that the ABO distribution in Saudi research follows this pattern: O>A>B>AB. While the number of people that are Rhesus (D) negative is insignificant [16]. Therefore, our finding in terms of blood group frequency among participants agrees with this study. As a result, Saudi Arabia's health authorities, as recommended before by another study [17], should be encouraged to work together to conduct bigger research comparable to that of [15,14], which followed individuals for thirty years. Our findings support another study that found unsatisfactory levels of protection amongst health care workers who received vaccination during childhood [25]. As our study targeted medical students who will be future health workers, this will maximize the benefits of preparing them to get protected just before commencing their careers. In conclusion, this work contributes to our knowledge and observations of anti-HBV immunity in people who were vaccinated as children twenty years ago. A substantial majority of students had a non-protective anti-HBs titer, according to our data. Booster dosages are highly recommended in order to trigger the memory immune response and provide protection against hepatitis B virus.

## Ethical Consideration:

The Najran university's Scientific Research Ethical committee approved the study procedures (SREC No.: 442-37-13718-DS). The study was conducted in December 2020.
